# Reduced Memory Coherence for Negative Events and Its Relationship to Posttraumatic Stress Disorder

**DOI:** 10.1177/0963721420917691

**Published:** 2020-06-04

**Authors:** James A. Bisby, Neil Burgess, Chris R. Brewin

**Affiliations:** 1 Division of Psychiatry, University College London; 2 Institute of Cognitive Neuroscience, University College London; 3 Institute of Neurology, University College London; 4 Department of Clinical, Educational and Health Psychology, University College London

**Keywords:** posttraumatic stress disorder, memory, flashbacks, coherence, hippocampus, amygdala

## Abstract

Posttraumatic stress disorder (PTSD) is characterized by disruptions in memory, including vivid sensory images of the trauma that are involuntarily reexperienced. However, the extent and nature of disruptions to deliberate memory for trauma remain controversial. A unitary account posits that all aspects of memory for a traumatic event are strengthened. In contrast, a dual-representation account proposes up-modulation of sensory and affective representations of the negative content and down-modulation of hippocampal representations of the context in which the event occurred. We take a neuroscientific approach and review the literature concerning the mechanisms required to produce coherent episodic memories and how they are affected in experiments involving negative content. We find, in healthy volunteers, that negative content can reduce associative binding and the coherence of episodic memories. Finally, we bring these findings together with the literature on PTSD to highlight how similar associative mechanisms are affected in patients, consistent with hippocampal impairment, supporting a dual-representation view of disrupted memory coherence.

Posttraumatic stress disorder (PTSD) is characterized by disruptive effects on memory, such as vivid sensory images of the trauma that involuntarily enter consciousness and are reexperienced in the present. In addition, disruption of deliberate recollection leading to fragmented and incoherent memories has been reported for traumatic events (Brewin, 2014). However, the observation that deliberate memories for traumatic events lack coherence—that is, different aspects are retrieved as individual fragments—remains controversial and has led to competing accounts.

In one view, memory phenomena in PTSD are explicable in terms of a unitary memory system for neutral and traumatic events, in which no special mechanism supports memory for the emotionally negative content of the latter (Rubin, Berntsen, & Bohni, 2008). This account suggests that emotional arousal during an event will enhance all aspects of memory (rather than disrupting it) via interactions between memory-related medial temporal lobe structures. Supporting this view, some studies comparing the trauma narratives of individuals with and without PTSD have failed to find differences despite using a variety of measures of fragmentation or coherence (Rubin et al., 2016). Thus, it has been argued that traumatic memories in PTSD are not fragmented and lacking in coherence (Engelhard, McNally, & van Schie, 2019; Rubin et al., 2008).

In contrast, the revised dual-representation theory of PTSD (Brewin, Gregory, Lipton, & Burgess, 2010) proposes that high levels of emotional arousal during an event strengthen lower-level imagery via up-modulation of the amygdala, whereas episodic memories subserved by the hippocampus are weakened. The imbalance between these systems increases intrusive imagery while impairing the coherence of voluntary recall. Evidence for these dissociable systems includes the observation that experimental manipulations, including alcohol administration, have different effects on intrusive images of a trauma film and on individuals’ ability to recognize and recall its content (Bisby, King, Brewin, Burgess, & Curran, 2010; Brewin, 2014)—findings that are robust to alternative explanations (Lau-Zhu, Henson, & Holmes, 2019).

In this article, we report a separate program of research that documents how, in nonclinical samples, the presence of negative content increases item encoding and decreases associative encoding. This mechanistic account of memory, derived from recent advances in cognitive neuroscience, sheds light on how traumatic experiences can affect different memory representations in opposing ways. Specifically, memory for the sensory-perceptual aspects of a negative event is strengthened, whereas memory for associations between the content and context in which the event took place is weakened, disrupting coherent episodic recall (Bisby & Burgess, 2017; Brewin et al., 2010). Here, we focus on (a) the role of the hippocampus in associative binding and memory coherence and (b) how these processes might be affected in PTSD.

## Mechanisms Supporting Coherent Memories

Episodic memories involve multiple pieces of information, such as the people, objects, and locations that make up an experience. When an individual retrieves an experience, all of the separate attended elements from the event are brought to mind, giving rise to the rich recollective experience that characterizes episodic retrieval. For this holistic recollective process to occur, the individual elements from an event must be bound together as a single memory representation, allowing for their complete reinstatement at retrieval.

The mechanisms supporting memory for the associations between the content of an experience and the context in which it occurred go far beyond memory for the content alone. Whereas memory for the individual elements is thought to be supported by neocortical areas such as perirhinal and parahippocampal cortices, binding them into a single representation relies on the hippocampus (Cohen & Eichenbaum, 1993). An important function of this associative binding is that the presentation of a partial input will reinstate all event elements via hippocampal pattern completion (Marr, 1971). Thus, the hippocampus is fundamental in supporting the associative structure of an event memory and in influencing how the memory is reexperienced at retrieval.

In line with a pattern-completion account, recent findings have shown that memories for previously experienced events are retrieved in a holistic manner. When participants learn multi-element events, associative retrievals from the same event are correlated, suggesting that events are stored and retrieved in an all-or-nothing way (Horner & Burgess, 2013). Accordingly, hippocampal activity at encoding predicts subsequent associative-memory performance and the binding of all attended event elements into a single representation. Further, hippocampal activity at retrieval supports reinstatement of associated elements in neocortical regions and not just the individual elements in question (Horner, Bisby, Bush, Lin, & Burgess, 2015). Therefore, hippocampal-dependent binding is fundamental to creating coherent memories, and the holistic retrieval of associated elements relies on hippocampal pattern completion and reinstatement of those elements in neocortical areas (even though pattern completion may be partial rather than 100% complete; e.g., Squire & Wixted, 2011).

## How Does Negative Content Affect Memory and Its Coherence?

An emotionally arousing experience is expected to capture attentional resources, facilitating perceptual processing and enhancing memory encoding (Phelps & LeDoux, 2005). Numerous studies have shown that negative items are remembered better than neutral items and with a greater degree of subjective recollection (Sharot & Yonelinas, 2008), consistent with proposals of a unitary view of memory in PTSD (Rubin et al., 2008). However, an important caveat is that this facilitation effect does not seem to affect associative binding in a similar way. That is, the presence of negative items at encoding can result in a reduction in subsequent memory for item–item and item–context associations (Bisby & Burgess, 2014), showing that negative experiences do not affect all aspects of memory in the same way.

The amygdala plays an important role in memory enhancements for negative events, facilitating perceptual processing (Phelps & LeDoux, 2005) to support emotion-to-content binding via perirhinal cortices (Yonelinas & Ritchey, 2015). Increased amygdala activity reliably predicts subsequent memory for emotional items (Kensinger & Schacter, 2006), but associative binding via the hippocampus is not always influenced in the same way (Ritchey, Wang, Yonelinas, & Ranganath, 2019).

Although the amygdala may facilitate aspects of emotional-memory formation, possibly via neuromodulation of the hippocampus, this mechanism cannot explain observations that item- and associative-memory encoding are often affected by emotion in opposing ways. Recent evidence shows that amygdala activity is increased during the presence of negative items, whereas hippocampal activity is reduced, coinciding with decreased associative-memory performance (Bisby, Horner, Horlyck, & Burgess, 2016).

Thus, the amygdala may inhibit hippocampal processing, either via neuromodulation or directly (Dolleman-Van der Weel, Lopez da Silva, & Witter, 1997). For example, reductions in item–context encoding occur following cortisol administration, even during early phases of the stress response (van Ast, Cornelisse, Meeter, Joels, & Kindt, 2016). Further, it has been proposed that interactions between glutamate and noradrenaline will mean that negative items within a scene are prioritized and strengthened, whereas associations with neutral items or context are weakened (Mather, Clewett, Sakaki, & Harley, 2016), although such a mechanism would not explain reduced associative memory between two negative items presented together.

If negative content impairs memory associations, it should reduce memory coherence. Bisby, Horner, Bush, and Burgess (2018) required participants to learn a series of events consisting of multiple elements (person, location, object). Coherence was tested by examining the pattern of multiple retrievals from each event to assess their relatedness. If memories are stored as bound representations, they should be retrieved in an all-or-nothing way, consistent with a pattern-completion process in which a partial cue triggers holistic retrieval of all event elements. In this study, although neutral events were stored and retrieved in a holistic way, a negative element at encoding (e.g., an injured person) reduced the relatedness of retrievals. That is, negative events were stored or retrieved in a less coherent way, suggesting impaired pattern completion.

## Memory Disruptions in Patients With PTSD

Consistent with memory being disrupted in PTSD, findings have shown anatomical alterations in memory-related brain structures. Structural abnormalities in PTSD typically involve volume reductions in a range of areas, including the hippocampus, ventromedial prefrontal cortex (vmPFC), and although less reliably, the amygdala (Logue et al., 2018). These anatomical differences in PTSD may be a result of the trauma exposure or a risk factor that is of genetic or environmental origin. Chronic stress can have detrimental effects on structures such as the hippocampus and vmPFC (Sapolsky, Uno, Rebert, & Finch, 1990). However, a study assessing identical twins discordant for combat exposure found reduced hippocampal volume in veterans with PTSD and in their combat-unexposed, non-PTSD twin (Gilbertson et al., 2002). This finding suggests that reduced hippocampal volume might serve as a pretrauma risk factor for PTSD.

Patients with PTSD demonstrate a number of memory deficits consistent with disruption of the machinery required to form coherent representations. Numerous studies have found that hippocampal-related tasks, such as memory for paired associates, are impaired in PTSD (Golier et al., 2002) and that reductions in associative memory are greater than item-memory disruptions (Guez et al., 2011). A deficit in hippocampal-prefrontal-thalamic circuitry, responsible for context processing and allowing organisms to disambiguate cues, has been proposed to underlie PTSD (Liberzon & Abelson, 2016).

Within the spatial domain, the hippocampus is implicated in allocentric-memory representations, the representation of locations of environmental features relative to each other. These contrast with egocentric memory, the representation of locations relative to the viewer (Burgess, Maguire, & O’Keefe, 2000). Using a configural learning task to test allocentric memory in twins discordant for combat trauma and PTSD, Gilbertson and colleagues (2007) found allocentric-memory reductions in both trauma-exposed PTSD patients and their unexposed twins. Memory decrements correlated with reductions in hippocampal volume, again highlighting the possibility that impairments might predate the traumatic episode and pose a risk factor. In another study using virtual reality to test spatial memory, patients with PTSD were found to display selective impairments in allocentric-memory processing, whereas egocentric-memory and item-memory performance were intact (Smith, Burgess, Brewin, & King, 2015).

Consistent with the idea that traumatic content does not simply strengthen episodic memories, recent findings from a study assessing memory in firefighters for emergency calls showed that memory for the events was impaired and that this reduction was linearly related to increases in stress (Metcalfe, Brezler, McNamara, Maletta, & Vuorre, 2019). Difficulties in deliberate recall might relate to a specific response, dissociation, that sometimes occurs during extreme stress. As well as being a risk factor for PTSD, dissociation interferes with memory encoding and is related to self-reported memory disorganization (Brewin, 2014). Dissociation might preferentially impact allocentric encoding, suggesting other aspects of memory performance whose relation to lack of coherence could be tested.

## Conclusions and Future Directions

In this review, we have outlined a current controversy concerning memory in PTSD and whether patients demonstrate impaired memory and a lack of coherence in their recall of the trauma. Although some researchers have claimed that such recall is not impaired in PTSD (Engelhard et al., 2019; Rubin et al.,2008, 2016), evidence from animal and human research demonstrates that high levels of stress can impair memory formation (Brewin, 2014; Jacobs & Nadel, 1985; Metcalfe et al., 2019). Here, we have detailed evidence that negative emotion can affect distinct aspects of memory in opposing ways in healthy volunteers, highlighting that such effects would not require a special mechanism specific to PTSD (Rubin et al., 2008). That is, negative emotion can strengthen negative content via amygdala up-modulation but disrupt hippocampal-dependent binding to weaken the associative structure of events and their later holistic recall via pattern completion (Bisby & Burgess, 2017; Brewin et al., 2010).

Although the salience of emotional items will likely attract greater processing, and this would contribute to the positive effects of emotion on item encoding, this process cannot fully account for disruptions in associative memory such as that between two negative items (see Bisby & Burgess, 2014; Bisby et al., 2016). From a translational perspective, we have discussed how neural structures supporting memory coherence are disrupted in PTSD, providing a basis for the way in which memory representations of traumatic and nontraumatic events might be altered in patients. Testable predictions include the idea that both facilitating hippocampal-dependent associative memory and reducing amygdala responsivity should reduce intrusive imagery in patients.

It is difficult to reconcile a view that proposes uniformly strengthened memory for the traumatic event (Engelhard et al., 2019; Rubin et al., 2008, 2016) with the evidence demonstrating impaired hippocampal function and reduced hippocampal volume in PTSD. If these structural abnormalities do highlight a potential risk factor for PTSD, an important question for unitary accounts is how a dysfunctional hippocampus can strengthen memory to result in coherent representations. It is also important to recognize that there is no single “trauma memory” but rather a series of memories corresponding to unfolding events (Brewin, 2016). In terms of the opposing effects that negative content can have on memory, differences in the response of an individual across the whole event might impact distinct representations for different periods of the traumatic event to a varying extent.

In conclusion, we have attempted to address a controversy in the functional interpretation of the symptoms of PTSD by reviewing the effects of negative emotion on episodic memory in healthy volunteers. We focused on the coherence of episodic memories generated by associative processing in the hippocampus that can support the process of pattern completion by which a partial cue can lead to reinstatement of all aspects of a memory. We have shown how negative events can disrupt these processes in healthy individuals and have argued that these mechanisms are impaired in PTSD, resulting in impaired binding and holistic retrieval in the disorder. Studies investigating such basic mechanisms provide a valuable addition to clinical studies of trauma narratives and will, in future, be crucial in providing further insight into memory disruptions in PTSD.

## Recommended Reading

Bisby, J. A., Horner, A. J., Bush, D., & Burgess, N. (2018). (See References). Shows how the experience of negative content can lead to a more fragmented event representation, reducing memory coherence and resulting in less holistic retrieval of related information.

Brewin, C. R., Gregory, J. D., Lipton, M., & Burgess, N. (2010). (See References). Presents a neural model of the way in which intrusive memories in posttraumatic stress disorder might develop through an imbalance between contextual and sensory representations.

Gilbertson, M. W., Williston, S. K., Paulus, L. A., Lasko, N. B., Gurvits, T. V., Shenton, M. E., . . . Orr, S. P. (2007). (See References). Examines allocentric memory in identical twins discordant for combat trauma and posttraumatic stress disorder (PTSD); shows that allocentric-memory deficits in individuals with PTSD are associated with decreases in hippocampal volume and that reductions in both were also seen in the nonexposed twin, highlighting a potential preexisting risk factor.

## Transparency

*Action Editor:* Randall W. Engle

*Editor:* Randall W. Engle
